# A novel immunodeficient NOD.SCID*-rd1* mouse model of retinitis pigmentosa to investigate potential therapeutics and pathogenesis of retinal degeneration

**DOI:** 10.1242/bio.021618

**Published:** 2017-03-03

**Authors:** Alaknanda Mishra, Barun Das, Madhu Nath, Srikanth Iyer, Ashwani Kesarwani, Jashdeep Bhattacharjee, Shailendra Arindkar, Preeti Sahay, Kshama Jain, Parul Sahu, Prakriti Sinha, Thirumurthy Velpandian, Perumal Nagarajan, Pramod Upadhyay

**Affiliations:** 1 Product Development Cell-1, National Institute of Immunology, Aruna Asaf Ali Marg, New Delhi 110067, India; 2 Department of Ocular Pharmacology, Dr. Rajendra Prasad Centre for Ophthalmic Sciences, All India Institute of Medical Sciences, New Delhi 110029, India

**Keywords:** Retinitis pigmentosa, NOD.SCID*-rd1* mouse model, Immunocompromised mouse, Cell based therapeutics

## Abstract

Retinitis pigmentosa (RP) is a common retinal degeneration disease caused by mutation in any gene of the photo transduction cascade and results in photoreceptor dystrophy. Over decades, several animal models have been used to address the need for the elucidation of effective therapeutics and factors regulating retinal degeneration to prohibit or renew the damaged retina. However, controversies over the immune privilege of retina during cell transplantation and the role of immune modulation during RP still remain largely uninvestigated because of the lack of suitable animal models. Here, we have developed an immunocompromised mouse model, NOD.SCID-*rd1*, for retinitis pigmentosa (RP) by crossing CBA/J and NOD SCID mice and selecting homozygous double mutant animals for further breeding. Characterization of the newly developed RP model indicates a similar retinal degeneration pattern as CBA/J, with a decreased apoptosis rate and rhodopsin loss. It also exhibits loss of T cells, B cells and NK cells. The NOD.SCID-*rd1* model is extremely useful for allogenic and xenogenic cell-based therapeutics, as indicated by the higher cell integration capacity post transplantation. We dissect the underlying role of the immune system in the progression of RP and the effect of immune deficiency on immune privilege of the eye using comparative qPCR studies of this model and the immune-competent RP model.

## Abstract (precis)

**Summary:** NOD.SCID-*rd1* is an immune compromised mouse model of retinitis pigmentosa (RP) to investigate cell-based therapeutics for retinal rescue during RP and to study immunological aspects of its pathogenesis and progression.

## INTRODUCTION

Retinal differentiation and maturation is a strictly regulated process in humans (Yang, 2004). The retinal degeneration diseases are irreversible once the retinal cells have degenerated because the adult retina is considered to lack stem cells and the cells lost are never regenerated (Jeon et al., 1998). To address this need, the recently emerging field of regenerative medicine seems to be promising where different sources of pluripotent and somatic cells are reprogrammed into a specific cell type and transplanted into the site of the defect (Bharti et al., 2014a; Ouyang et al., 2016; Siqueira, 2011). Although these studies remain in the initial phase, it is expected that this may open newer therapeutic options for the retinal degeneration diseases. Over many decades, animal models have been frequently used to elucidate the factors regulating retinal degeneration and to develop ways to prohibit or renew the damaged retina. Researchers have also used a variety of retinal degeneration models according to the purpose of their study (Chang et al., 2002; Chang, 2013; Veleri et al., 2015).

The *Pde6b rd1* mouse model is one of the successfully used and widely characterized mouse models for retinitis pigmentosa (Chang, 2013; Veleri et al., 2015). It shows an early onset of retinal degeneration starting from weaning age due to a xenotropic murine leukemia viral insert (Xmv28) in the first intron of *Pde6b* and a non specific mutation in the 349th base pair of exon 7 of the *Pde6b* gene (Chang, 2013). The *Pde6b* gene encodes rod cGMP-specific 3′, 5′-cyclic phosphodiesterase subunit-β.

Since the eye is also considered to be an immune privileged site, there has been a trend to use immune competent mouse models for cell-based transplantation studies (Masli and Vega, 2011; Taylor, 2016).While the immune privilege stands true for some instances, mostly for the anterior chamber of the eye, it is not an absolute phenomenon and its mechanisms still remain poorly dissected (Forrester and Xu, 2012; Hori et al., 2010; Taylor, 2016).There is also the risk of immune cell penetration towards the posterior chamber of the eye as the blood-retinal barrier loses its integrity due to loss of photoreceptor and retinal pigment epithelial (RPE) cells, which can lead to immune rejection or immune cell-targeted loss of transplanted cells (Forrester and Xu, 2012; Xian and Huang, 2015a).The ability of adaptive and innate immune reactions to weaken engraftment of stem cell transplants is an important aspect of the host reaction that can affect the efficiency of cell transplantation (Cibelli et al., 2013).

Although a lot has already been proposed about the pathogenesis of the disease (Berson et al., 2002; Camacho and Wirkus, 2013; Chang et al., 2002; Chang, 2013; Veleri et al., 2015; Wright et al., 2010), little is known about the role of immune system in the progression of RP as it is mainly considered to be a hereditary disease. Alterations in retinal homeostasis secondary to aging, metabolic abnormalities, altered vascular perfusion or degenerative genetic conditions may initiate various inflammatory cascades that result from the breaching of the posterior eye compartment due to breakdown of the blood-retinal barrier that sheaths the ocular environment from an immune response (Forrester and Xu, 2012; Hori et al., 2010; Whitcup et al., 2013).

Moreover, it is of further importance to dissect out the part of immune system that is involved in degeneration and inflammation. Not much is known of the individual effects of adaptive or innate immunity in retinal degeneration and progression during RP. The evaluation of such conditions may, however, become restricted due to unavailability of animal models that mimic the condition in which immune cells are absent so that a proper comparison of disease progression may be devised.

Hence, in our present study, we developed an immunocompromised mouse model of RP lacking in the function of *Pde6b* (which functions in phototransduction cascade) and *Prkdc* (which encodes the catalytic subunit of the DNA-dependent protein kinase, DNA-PK). The homozygous *Pde6b^−/−^ Prkdc^−/−^* mouse model was named as NOD.SCID-*rd1* where NOD.SCID indicates lack of T, B and NKT cells and *rd1* stands for *Pde6b*^−/−^ retinal degeneration model. The proposed model mimics the diseased condition (RP) making it more apt for analyzing the role of immune cells in retinal degeneration and the pros and cons of the cell-based therapeutics.

## RESULTS

### Hematology

**Fig. 1.**
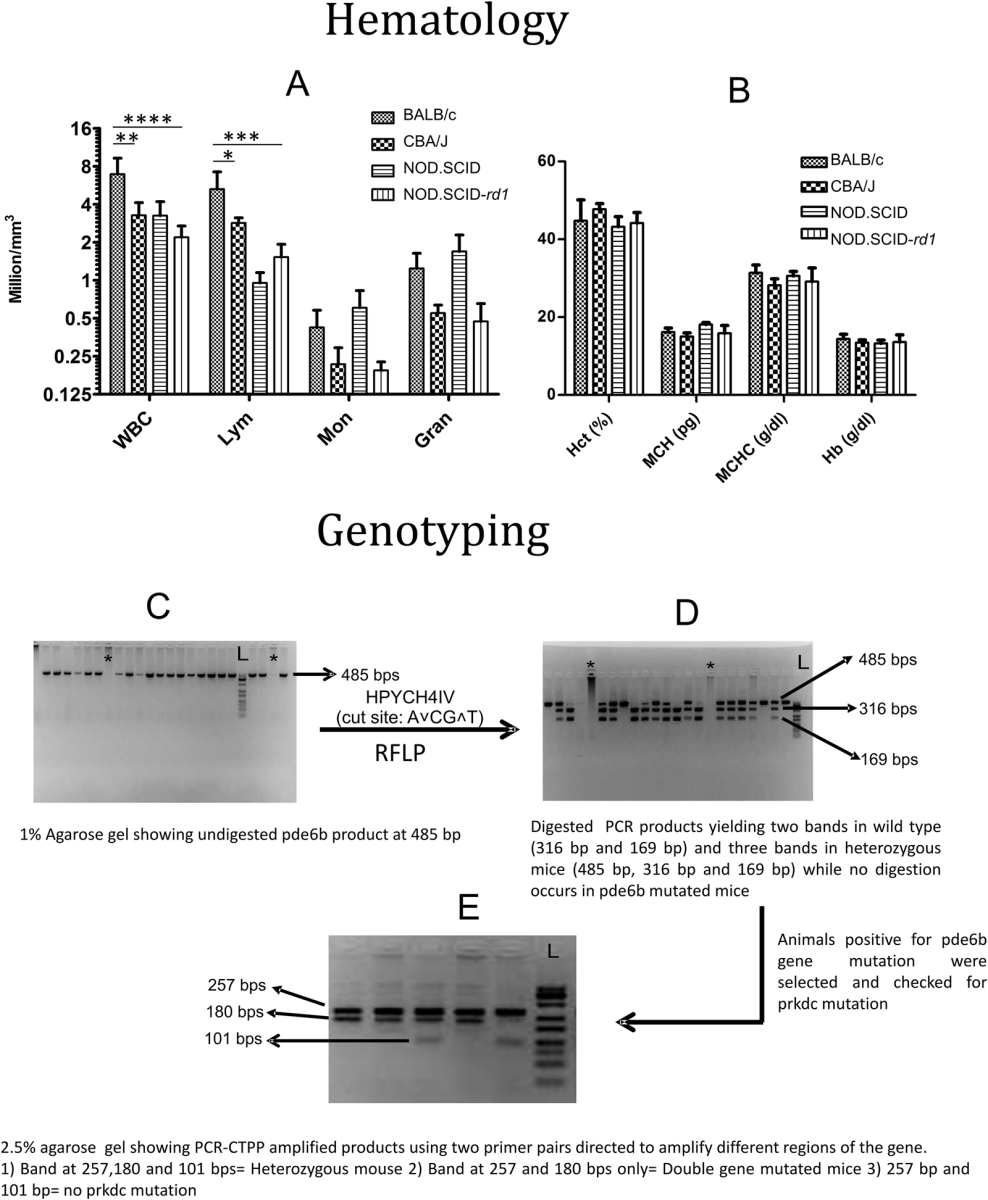
**Hematological analysis and genotyping for NOD.SCID-*rd1* model of RP.** (A,B) Graphical representation of comparative analysis for hematological parameters amongst BALB/c, CBA/J and NOD.SCID-*rd*1 strains of mice (*n*=8). The result indicates that WBC and lymphocyte proportion of CBA/J and NOD.SCID-*rd1* mice is reduced compared to BALB/c; however, there is a drastic reduction in WBC and lymphocyte proportion in NOD.SCID-*rd1* mice. Moreover, NOD SCID mice also show an increased monocyte and granulocyte proportion while both CBA/J and NOD.SCID-*rd1* exhibit decreased levels of monocytes and granulocytes instead of any elevation. Other parameters like Hct, MCH, MCHC and Hb showed a similar trend in all the strains of mice. (****P<0.0001, ***P≤0.001, **P<0.01and *P≤0.05). (C,D) Gels showing amplified *Pde6b* product at 485 bp and HPYCH4IV digested *Pde6b* amplicons respectively. RFLP analysis yields two bands (316 bp and 169 bp) for the wild type, and three bands (485 bp, 316 bp, 169 bp) for heterozygous animals. HypCH4IV, whose cutting site is A^∨^CG_∧_T, failed to digest *Pde6b* mutated amplicons, which undergo single nucleotide mutation in 349th base pair of exon 7. (E) The animals testing positive for Pde6b mutation were further analyzed for *Prkdc* mutation (which leads to B- and T-cell dysfunction) by PCR-CTPP method. The *Prkdc* mutated gene amplicons yielded bands at 257 and 180 bp, heterozygous animals at 257 bp, 180 bp and 101 bp, where bands were 257 bp and 101 bp in wild-type animals.

All the hematological parameters of NOD.SCID-*rd1* mice were comparable to CBA/J mice except total leukocytes and lymphocytes, which were significantly lower in NOD.SCID-*rd1* compared with BALB/c and CBA/J (Fig. 1A). However, compared to the NOD SCID mice, it showed no significant changes in the proportion of leukocytes and lymphocyte or any other parameters, such as hemoglobin, MCH and MCHC (Fig. 1B).

### Genotyping

#### *Pde6b* mutation screening

*Pde6b* gene amplification resulted in a 485 bp product in wild-type and *Pde6b*-mutated animals (Fig. 1C). The product was further digested by HpyCH4IV restriction enzyme (cut site A^∨^CG_∧_T). Since a nonsense mutation at base pair 349 of exon 7 in *Pde6b* (C to A) changes the sequence to AAGT, the PCR product of the mutated animal is not digested, unlike the wild-type sequence. Hence, upon digestion, the wild-type gene gives a 316 bp and 169 bp fragment while the heterozygote gives three fragments each of 485 bp, 316 bp and 169 bp and mutated gene is not digested at all (Fig. 1D).

#### *Prkdc* mutation screening

Genotyping of SCID mice (*Prkdc* gene mutated) was done by allele-specific polymerase chain reaction with confronting two pair primers (PCR–CTPP) assay. Here, mutation causes a T to A single nucleotide transversion in exon 85 of the *Prkdc* gene. This method of genotyping employs amplification of different regions of gene for mutated and wild-type alleles.

The SCID homozygous gene yields two products at 257 bp and 180 bp, the wild-type gene shows two fragments at 257 bp and 101 bp while heterozygotes show three bands at 257 bp, 180 bp and 101 bp owing to one mutated and one wild-type allele (Fig. 1E).

### Immune cell analysis in NOD.SCID *rd1* mice

**Fig. 2.**
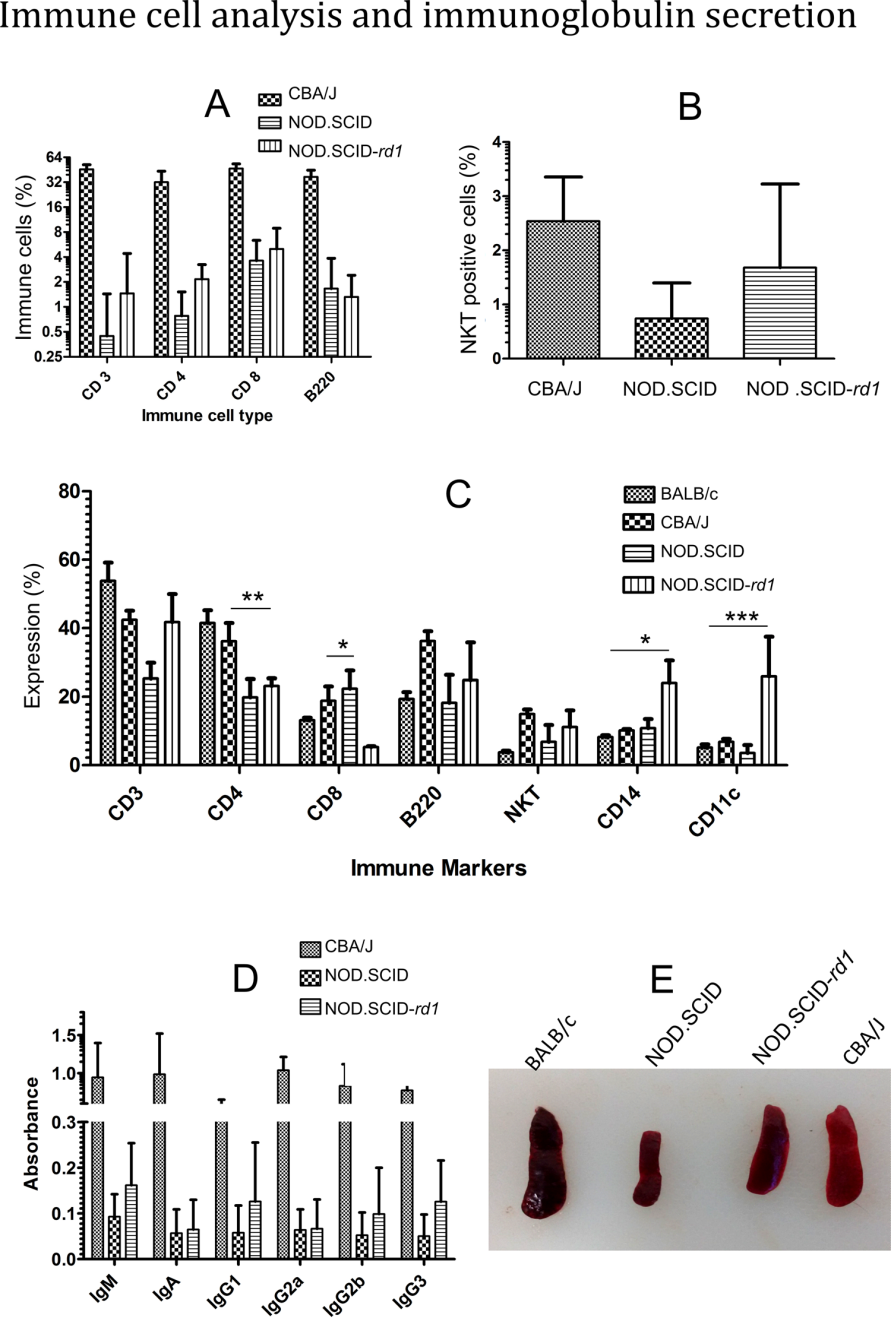
**Immune cell analysis and relative immunoglobulin quantification.** Graphical representation of flow cytometric analysis of immune cell markers in (A,B) peripheral blood (*n*=15) and (C) spleen of CBA/J, NOD.SCID-*rd1* and NOD SCID strains of mice (*n*=5). The peripheral blood analysis revealed that there is no significant difference in the level of CD3^+,^ CD4^+^, CD8^+^, B220^+^ and NKT^+^ cells between NOD.SCID-*rd1* and NOD SCID, suggesting immunocompromised nature of NOD.SCID-*rd1*. Moreover, the CD4^+^ and CD8^+^ T cells in NOD.SCID-*rd1* spleen were significantly reduced compared with CBA/J while showing a drastic increase in CD14^+^ and CD11c^+^ cell population. (D) Relative quantification of immunoglobulin secretion for CBA/J, NOD SCID and NOD.SCID-*rd1* through ELISA (*n*=10). The result indicated no significant difference between NOD.SCID-*rd1* and NOD SCID immunoglobulin levels. (E) Representative images showing spleen size in BALB/c, CBA/J, NOD.SCID-*rd1*and NOD SCID, respectively. The BALB/c mouse had largest spleen size while NOD SCID had the smallest spleen. CBA/J, however, had reduced size of spleen compared with BALB/c while NOD.SCID-*rd1* has an intermediate size of spleen lying between CBA/J and NOD SCID (parent strains). ***P≤0.001, **P<0.01 and *P≤0.05.

The levels of CD3^+^, CD4^+^, and CD8^+^, B220^+^ and NKT^+^ cells were evaluated in peripheral blood and spleen of NOD.SCID-*rd1* compared to CBA/J and NOD SCID. It was observed that there was no significant difference in the expression of any of these cells between NOD.SCID-*rd1* and NOD SCID while CBA/J showed a significantly higher proportion of CD3^+^, CD4^+^, CD8^+^ and B220^+^ cells as compared to both NOD SCID and NOD.SCID-*rd1* in peripheral blood. Surprisingly, the NKT^+^ cell levels of CBA/J mice were greatly reduced in comparison to normal vision strains like BALB/c. However, NOD SCID mice showed an even lower proportion of NKT^+^ cells while levels remained similar for CBA/J and NOD.SCID-*rd1*(Fig. 2A,B).

Immune cell analysis in the spleen revealed that CD3^+^, CD4^+^, CD8^+^ and B220^+^ cell populations in NOD SCID mice were greatly reduced. While CD3^+^ cells showed no significant difference in NOD.SCID *rd1* compared with CBA/J, CD4^+^ and CD8^+^ cells were distinctly reduced in NOD.SCID *rd1*, indicating extreme loss of cytotoxic and helper T cells. B220^+^ and CD8^+^ cells showed an upregulation in CBA/J which may point towards the role of self or auto antibody production during RP, which further progresses retinal degeneration condition. However, the NOD.SCID-*rd1* mice displayed levels of B220^+^ cells comparable to NOD SCID and significantly lower than CBA/J. NKT^+^ cells remained without any significant change in both CBA/J and NOD.SCID-*rd1*. The NOD.SCID-*rd1* mice exhibited extremely higher levels of CD14^+^ (marker for macrophages) and CD11c^+^ (marker for dendritic cells) cells, the major innate immunity components, compared with CBA/J. The result also suggests that B cells and cytotoxic T cells (CD8^+^) have a greater role to play during retinal degeneration owing to their extremely increased expression in CBA/J (Fig. 2C).

### Relative quantification of immunoglobulin secretion

Immunoglobulin levels of NOD.SCID-*rd1* (IgA, M, G1, G2a, G2b and G3) were quantified relative to NOD SCID and CBA/J mice. The result suggested that there was no significant difference between immunoglobulin levels of NOD SCID and NOD.SCID-*rd1* mice; however immunoglobulin levels of CBA/J were significantly higher than in both the strains. This indicates that B cells are mostly absent in NOD.SCID-*rd1* mice as in NOD SCID, and are non functional even if a small percentage of B cells remained (Fig. 2D).

### Qualitative analysis of spleen

The spleen size of NOD SCID was the least amongst all the strains while BALB/c exhibited largest spleen size. NOD.SCID-*rd1* had an intermediate size to that of CBA/J and NOD SCID (Fig. 2E).

### Quantitative RT-PCR analysis for retina-specific genes

**Fig. 3.**
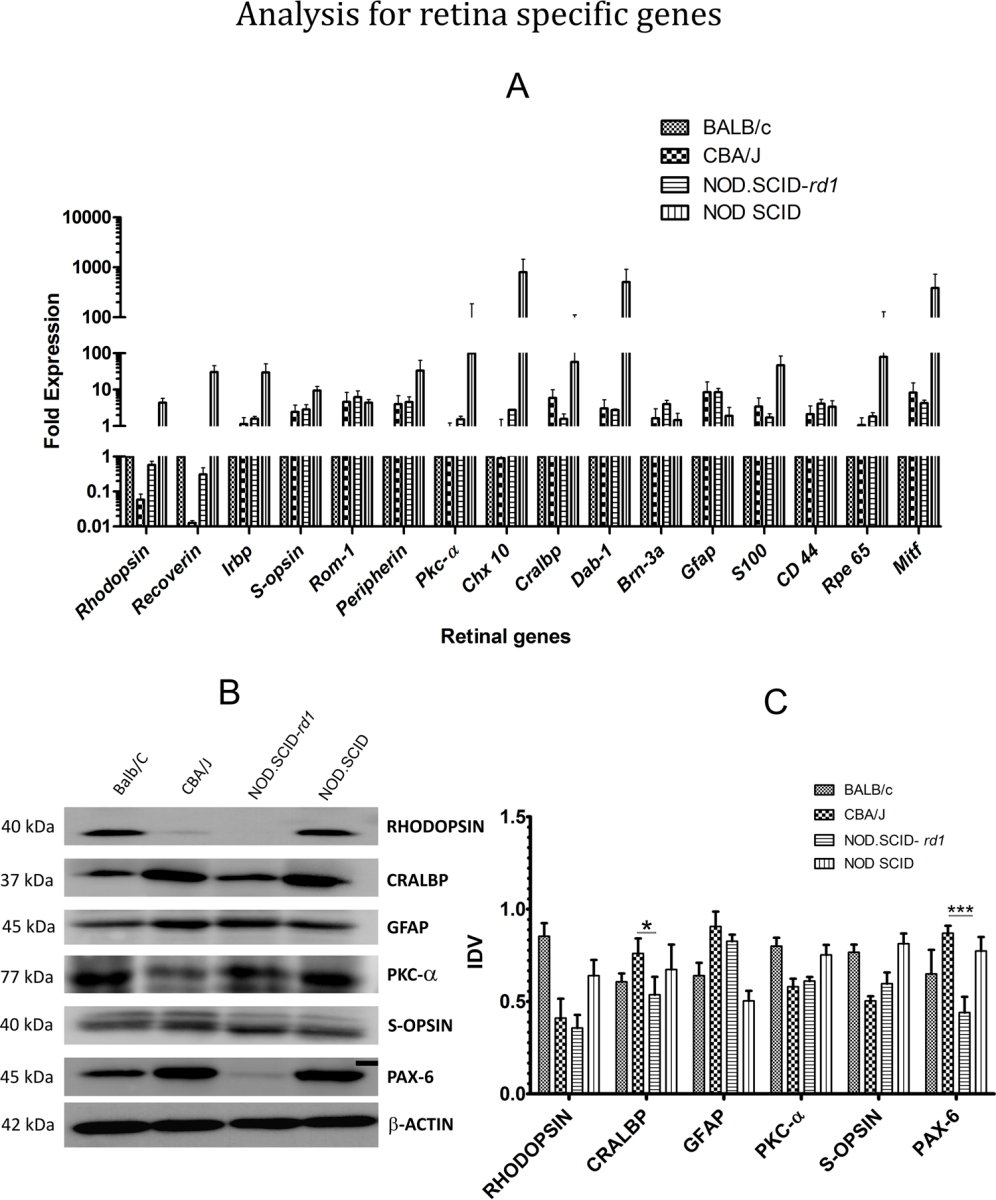
**Analysis of retinal-cell-specific transcripts and protein levels.** (A) Relative quantification of retinal cell transcripts in CBA/J and NOD.SCID-*rd1* mice strain, compared with that of Balb/C at 4 weeks of age. The rod PR markers [*Rho* (*Rhodopsin*) and *Rcvrn* (*Recoverin*)] were highly downregulated in CBA/J, while NOD.SCID-*rd1* showed a median expression level in between that of CBA/J and BALB/c. *Irbp* (interphotoreceptor retinoid-binding protein) expression remained similar in both CBA/J and NOD.SCID-*rd1*. Cone photoreceptor markers [*S-opsin*, *Rom1*, *Prph* (*Peripherin*)] remained comparable in CBA/J and NOD.SCID-*rd1* and exhibited higher expression thanBALB/c. Retinal Müller glial cell markers (*Gfap*, *S100* and *Cd44*) exhibited an upregulated expression in both CBA/J and NOD.SCID-*rd1* as against BALB/c. RPE cell marker (*Rpe65*) remained unaffected while *Mitf* showed an elevated expression in both CBA/J and NOD.SCID-*rd1*. Amacrine cell marker (*Cralbp*) was slightly upregulated in NOD.SCID-*rd1* and showed a highly elevated expression in CBA/J while *Dab1* showed a comparable increase in both CBA/J and NOD.SCID-rd1 as against BALB/c. NOD.SCID-*rd1* displayed an appreciative increase in the expression of retinal ganglion cell marker (*Brn3a*). CBA/J, however, indicated a marginal expression change as compared to BALB/c. Changes in the expression of retinal bipolar cell markers (*Pkca* and *Chx10*) were negligible in both CBA/J and NOD.SCID-*rd1*). (B) Representative images of western blot analysis for retinal proteins. (C) Integrated densitometry values (IDVs) indicated that CRALBP and PAX6 showed a lower expression in NOD.SCID-*rd1* as compared to CBA/J, while both these strains exhibited slight downregulation in the expression of rod PR marker (Rhodopsin), cone PR marker (S-opsin) and bipolar cell marker (PKC-α) as compared to BALB/c. Moreover, the expression of GFAP was highly upregulated in both CBA/J and NOD.SCID-*rd1* agreeing with the q-PCR results. ***P≤0.001 and *P≤0.05.

The mRNA levels of rod-specific genes were significantly reduced in CBA/J as compared to BALB/c (normal vision phenotype). NOD.SCID-*rd1*, however, shows an intermediate level of *Rhodopsin* and *Recoverin* expression demonstrating that NOD.SCID-*rd1*undergoes rod cell degeneration to a lesser extent than CBA/J*. Irbp*, found in the interphotoreceptor matrix of the retina between the retinal pigment epithelium and the photoreceptor cells, does not show any change in expression, suggesting that retinoid transport remains unaffected during RP. Moreover, cone photoreceptor markers (*S-opsin*, *Rom1* and *Prph*) and bipolar cell markers (*Pkca* and *Chx10*) show unaltered expression in both CBA/J and NOD.SCID-*rd1* with respect to BALB/c suggesting that these retinal cell types do not get affected by rod cell degeneration at the initial stages. CBA/J and NOD.SCID-*rd1* also exhibit higher mRNA levels in amacrine cell markers (*Cralbp*, *Dab1*) and retinal microglia markers (*Gfap*, *S100* and *Cd44*), suggesting an over-active cytokine/chemokine pathway and an increased gliotic index during RP. The retinal pigment epithelium (RPE) marker (*Rpe65*) remained unaffected, while *Mitf* showed an elevated expression in both CBA/J and NOD.SCID-*rd1*. The retinal ganglion cell (RGC) marker (*Brn3a*) was highly upregulated in NOD.SCID-rd1 while a slightly increased expression in CBA/J was also noticed. This suggests that ganglion cells, which are the only output cells of the retina, are well preserved and even upregulate during RP to compensate for the vision loss by enhanced output of signals to the brain (Fig. 3A).

### Western immunoblotting analysis

The protein levels of the retina-specific markers were checked by western blotting. The results obtained convey that rod-specific marker (Rhodopsin) protein expression was absent while the expression levels of cone specific marker (S-opsin) was unaffected as compared with BALB/c at the same age (4 weeks) in both CBA/J and NOD.ACID-*rd1*. The rod bipolar cell marker (PKC-α) showed attenuated expression in CBA/J, confirming its simultaneous but slower degeneration with rod cells while NOD.SCID-*rd1* exhibited normal expression of PKC-α. PAX6, a common retinal progenitor cell marker showed increased expression in CBA/J and attenuated expression in NOD.SCID-*rd1* compared with BALB/c. Nevertheless, CRALBP and GFAP, which are markers of amacrine cells and Müller glial cells respectively, were highly upregulated suggesting that they might be involved in the exacerbation of retinal degeneration during RP (Fig. 3B,C).

### Immunofluorescence analysis of photoreceptor markers

**Fig. 4.**
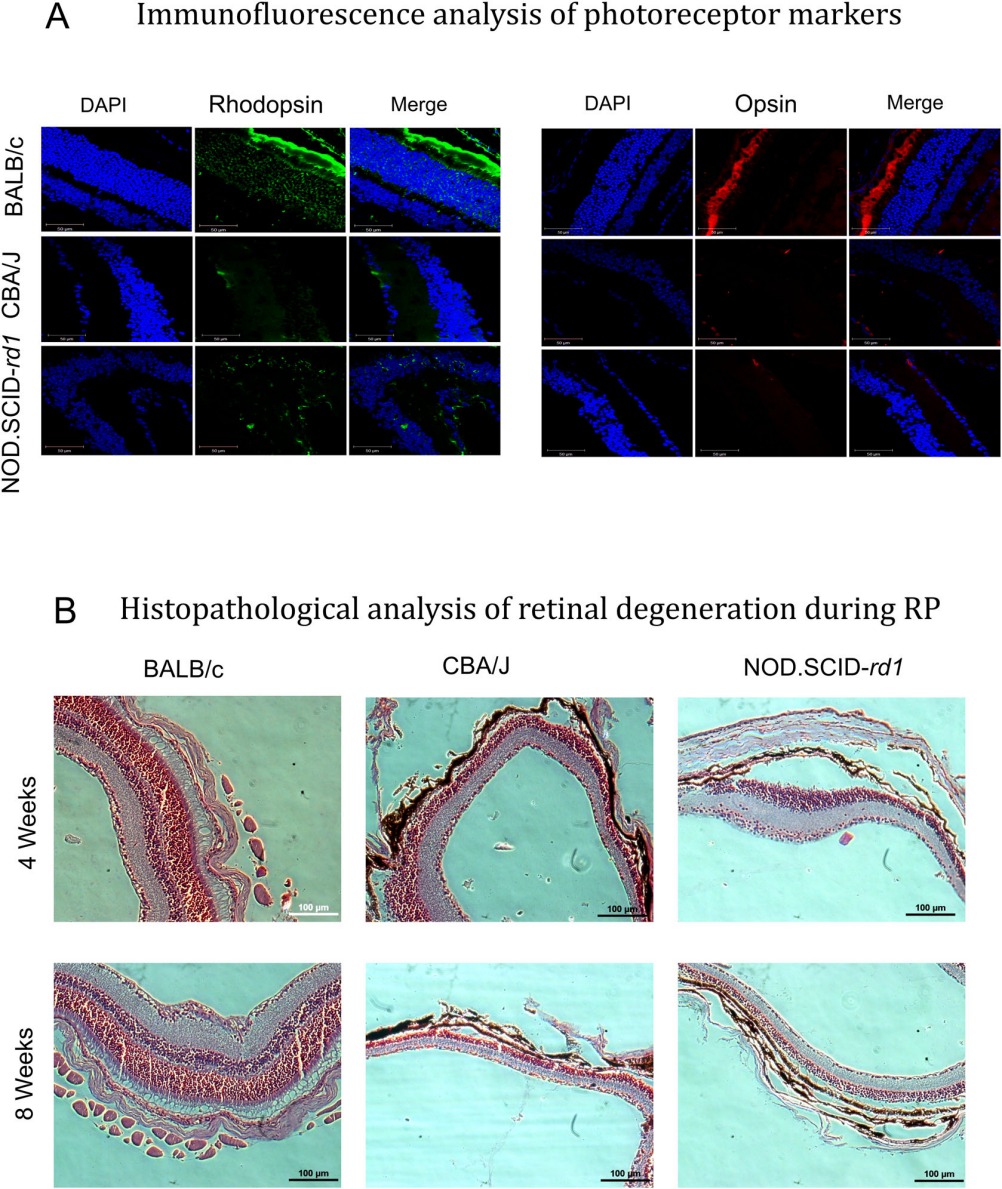
**Immunofluorescence analysis of photoreceptor markers and histopathological changes during retinal degeneration.** (A) Representative confocal micrographs (63×) for immunostaining of rod PR marker (Rhodopsin) and cone PR marker (S-opsin) in BALB/c, CBA/J and NOD.SCID-*rd1* at 4-6 weeks of age. BALB/c strain with normal vision showed normal rod and cone PR staining in the outer segment of retina while rd1 model CBA/J had completely degenerated outer segments. NOD.SCID-*rd1* also displayed outer segment degeneration, however, they had a few rhodopsin- and opsin-stained cells in the inner nuclear layer. The green color represents Rhodopsin-stained cells and red color represents S-opsin-stained cells in the retina. (B) Representative images illustrating histopathological analysis of retinal degeneration during RP in CBA/J and NOD.SCID-*rd1* compared with BALB/c at 4 weeks and 8 weeks of age. The outer segment and outer nuclear layer had degenerated with detachment from RPE layer at various points in both CBA/J and NOD.SCID-*rd1* by 4 weeks of age, with further thinning of inner nuclear layer by 8 weeks of age. BALB/c however retained intact retinal structure throughout.

Analysis for rod cell markers and cone cell markers was done to compare the level of degeneration of photoreceptor cells in CBA/J and NOD.SCID-*rd1*. It was observed that BALB/c (a normal vision control) exhibited proper outer segment staining for rods and cones while there was no staining observed in CBA/J, and NOD.SCID-*rd1* showed fewer cells expressing rod and cone markers in the inner nuclear layer (INL) and ganglion cell layer (GCL) of the retina. Müller cells, which act as retinal progenitors during degeneration could be the reason behind the low expression of these photoreceptor markers in degenerated retina in NOD.SCID-*rd1* (Fig. 4A).

### Histopathological analysis of retinal degeneration during RP

Histological analysis of the retina was performed by Hematoxylin and Eosin staining. BALB/c mice were used as a normal vision control. The CBA/J and NOD.SCID-*rd1* animals exhibited complete loss of outer segment (OS), photoreceptor (PR) layer and outer nuclear layer (ONL) at 4 weeks of age. However, the INL and GCL remained intact. This indicates that *Pde6b* mutation affects not only rod photoreceptor cells but also cell types in close contact to them, namely cone photoreceptor cells, bipolar cells or horizontal cells. At the age of 8 weeks, the density of the remaining INL reduces further, suggesting a time-dependent progression of retinal degeneration and cell apoptosis in both CBA/J and NOD.SCID-*rd1*(Fig. 4B).

### Fundoscopic retinal imaging

**Fig. 5.**
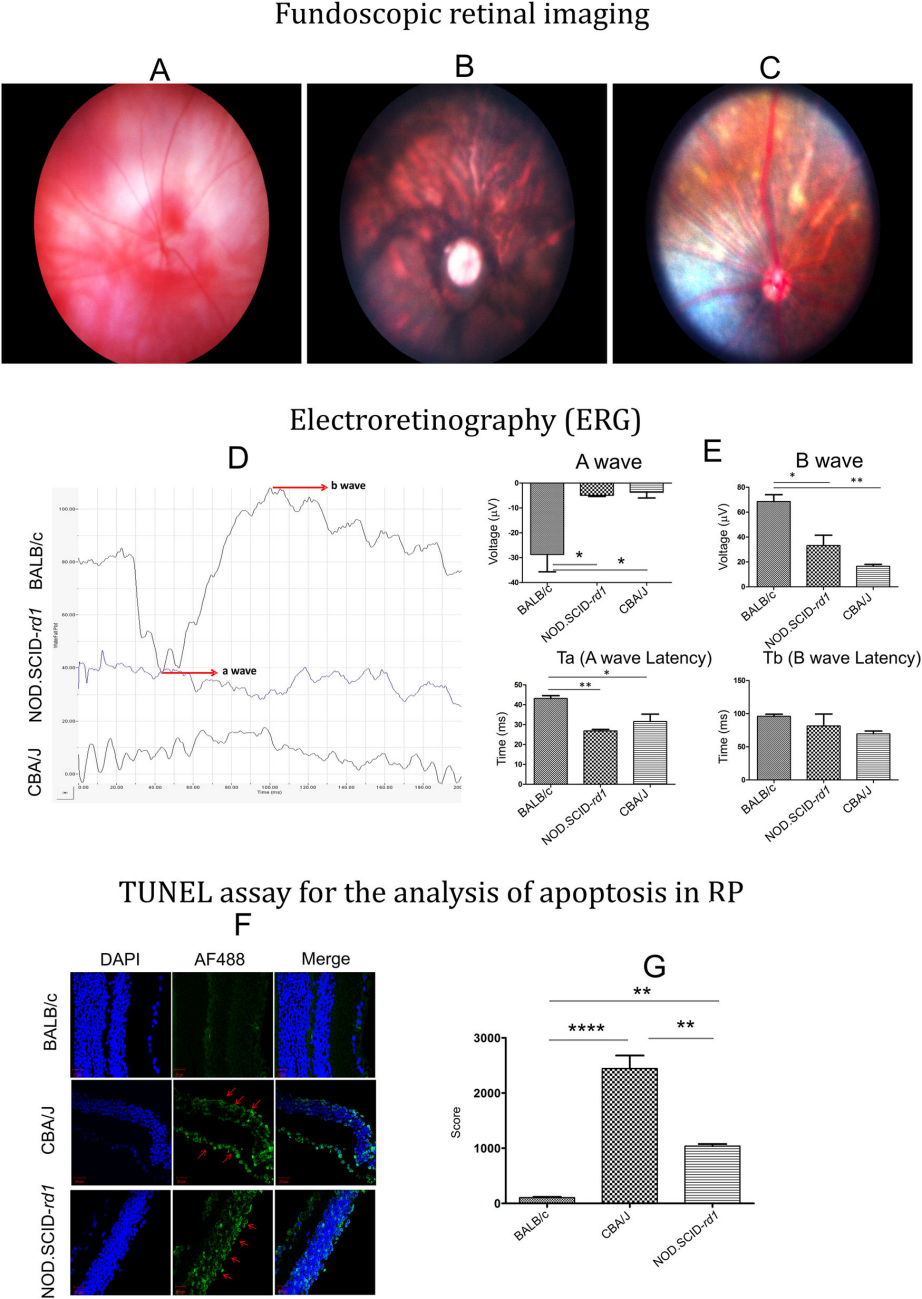
**Fundoscopic imaging, ERG analysis of retina and apoptosis during RP.** (A-C) Representative fundoscopic retinal images for the analysis of retinal degeneration and vessel attenuation in BALB/c, CBA/J and NOD.SCID-*rd1*, respectively at 4 weeks of age. Both CBA/J and NOD.SCID-*rd1* showed patched retina, however, the severity of attenuated vessels and patchy appearance was aggravated in CBA/J as compared to NOD.SCID-*rd1*. BALB/c, which had normal vision, exhibited no patches and clear red vessels were visible throughout retina. (D,E) Electroretinogram analysis of BALB/c, CBA/J and NOD.SCID-*rd1* at 4 weeks of age. BALB/c exhibited distinct a- and b-wave peaks while NOD.SCID-*rd1* and CBA/J had no a- and b-wave peaks with reduced a-wave latency indicating the degeneration of photoreceptors (rods and cones) as well as improper functionality of retinal bipolar cells. (F,G) Representative images illustrating apoptosis during retinal degeneration in CBA/J and NOD.SCID-*rd1* as compared to normal vision BALB/c. Ten random fields were scored for apoptotic cells in each case and graphically plotted. The result shows an extremely high apoptotic rate of retinal cells in CBA/J. While NOD.SCID-*rd1* also shows apoptosis, it still remained significantly lower than CBA/J. The green color represents TUNEL-positive Alexa Fluor 488-stained apoptotic cells. Nuclei are stained blue with DAPI.

Ocular fundus examination exhibited a large diversity between the strains observed. The albino mice, like BALB/c, displayed a much lighter, red-shining fundus, due to the lack of pigmentation. The fundus was devoid of any patchy structures or lobules, whereas CBA/J mice showed a patchy fundus with thick attenuated and sclerotic retinal vessels. The NOD.SCID-*rd1* mice on the other hand had less prominent patches and a lower degree of vessel attenuation than CBA/J (Fig. 5A-C).

### Electroretinography (ERG)

The retinal function was tested by ERG recorded at an illumination intensity of 1 cds/m^2^. The a- and b-waves were measured along with their latencies. The a-wave amplitude was defined as the difference between the amplitude at the onset of the stimulus to the maximum negative peak amplitude on averaged ERG recordings. The b-wave was measured from this negative peak to the next positive peak maximum. The latency of the a- and b-waves was measured from the onset of the light stimulus to the respective negative and positive peaks of the a- and b-waves. The NOD.SCID-*rd1* and CBA/J mice showed no response to the given flashlight stimuli, whereas for BALB/c mice, well developed a- and b-waves were recorded, indicating a normal retinal function. NOD.SCID-*rd1*showed similar pattern of a- and b-waves to CBA/J, re-confirming the complete loss of photoreceptors and bipolar cells at the age of 4 weeks (Fig. 5D,E).

### TUNEL assay for the analysis of apoptosis in RP

The retina was analyzed for apoptotic cells at 4 weeks of age for BALB/c, CBA/J and NOD.SCID-*rd1* to observe the effect of photoreceptor degeneration on the viability of other retinal cell types. Photoreceptor degeneration during RP results in a population of retinal cells in the INL that are highly prone to apoptosis, which demonstrates that the retina works as a unit and photoreceptors play central role in the phototransduction cascade; any damage or loss to these cells not only affects the integration of retina but also the functionality and viability of other retinal cells working in close proximity to PR cells. However, apoptosis rate was higher in CBA/J than NOD.SCID-*rd1* and showed a highly significant number of early apoptotic cells in the INL and a few in GCL as compared with BALB/c mice of the same age (Fig. 5F,G).

### Behavioral analysis

#### Visual cliff test

**Fig. 6.**
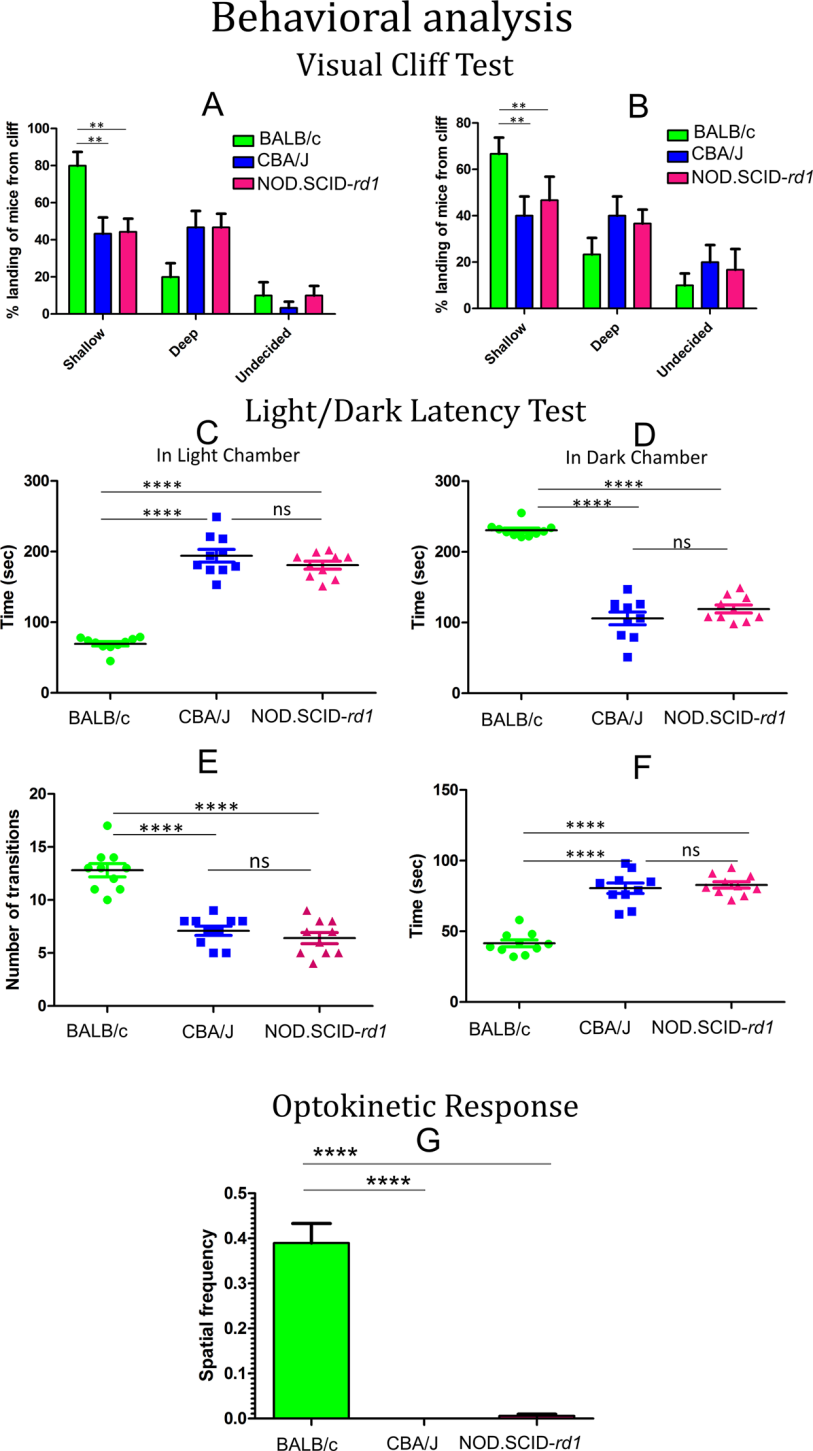
**Behavioral analysis of NOD.SCID-*rd1* model of RP.** (A,B) Behavioral analysis (*n*=10) by visual cliff test in bright (A) as well as dim (B) light showing that BALB/c mice preferred the shallow side of the cliff while both CBA/J and NOD.SCID-*rd1* exhibited a 50% tendency to step on either side of the cliff. (C,D) Light/dark latency test analysis indicated non-aversive behavior of CBA/J and NOD.SCID-*rd1* towards light that tended to spend more time in light chamber where they were initially placed. However, BALB/c mice were aversive to light and tend to spend more time in dark chamber. (E,F) The exploratory behavior of CBA/J and NOD.SCID-*rd1* as indicated by the number of transitions made between both chambers as well as the delay in time taken to enter dark chamber from light chamber, is also reduced compared with BALB/c. (G) The spatial frequency was calculated based on the optokinetic response of mice to various frequencies of gyrating stripes wherein CBA/J and NOD.SCID-*rd1* failed to show any response. ****P<0.0001 and **P<0.01.

The visual cliff test performed for the analysis of depth perception in mice revealed that CBA/J and NOD.SCID-*rd1* exhibited a 50% probability of stepping to either side (shallow or deep) of the raised platform. This can be explained by the visual defect in these mice which causes a lack of depth perception. The light intensity, i.e. dim (50 lux) or brightly lit (250 lux) conditions, does not affect the result in NOD.SCID-*rd1* or CBA/J. However, BALB/c mice, which have normal vision, show an inclination towards stepping to the shallow side of the platform both in dim and brightly lit conditions. The most well developed depth perception is noted at the age of 4-6 weeks when the percentage of stepping to the shallow side is higher in brightly lit conditions than dim conditions (Fig. 6A,B).

#### Light/dark latency test

In the light/dark latency test, the mice were placed in a white area (which they found aversive) and would generally move around the periphery until they found an opening, at floor level, to enable access to the black compartment, and this usually occurred within 30-50 s in BALB/c and 60-90 s in CBA/J and NOD.SCID-*rd1*. The essential feature was the measurement of increased transitions between the light and dark chambers and the time spent in each compartment. These criteria were measured and compared amongst BALB/c, CBA/J and NOD.SCID-*rd1* mice. Since mice are nocturnal animals, they prefer staying in the dark compartment. BALB/c mice when placed in the light compartment immediately moved into the dark compartment after finding the passage and spent a much more extended time in the dark chamber. However, in case of CBA/J and NOD.SCID-*rd1*, the mice took a longer time to find the passage to the dark chamber and also seemed to spend more or equal time in the light chamber. Moreover, the risk-taking behavior in these mice was comparatively lower as they did not like to enter the dark chamber at once (Fig. 6C-F).

#### Optokinetic response

The OKR test was conducted to observe optokinetic nystagmus through head-tracking movement during continuous rotation or movement. It was further observed that CBA/J and NOD.SCID-*rd1* showed minimal response to the optokinetic drum rotation. A continued decrease in optokinetic nystagmus (OKN) was found with increasing grating frequencies of stripes (0.03, 0.13, 0.26, 0.52 and 1.25 cpd), indicating that the visually disabled mice were unable to distinguish between two nearly located points i.e. they were lacking in OKN. The highest spatial frequency eliciting a response was taken as the maximum visual acuity limit in the mice (Fig. 6G). Interestingly, BALB/c which were used as a normal vision control also exhibited very poor OKN, which may further be explained by reports that suggest that BALB/c mice suffer from disturbances of the central visual system (Puk et al., 2008; Thomas et al., 2004).

### Effect of immune deficiency over immune privilege in eye

**Fig. 7.**
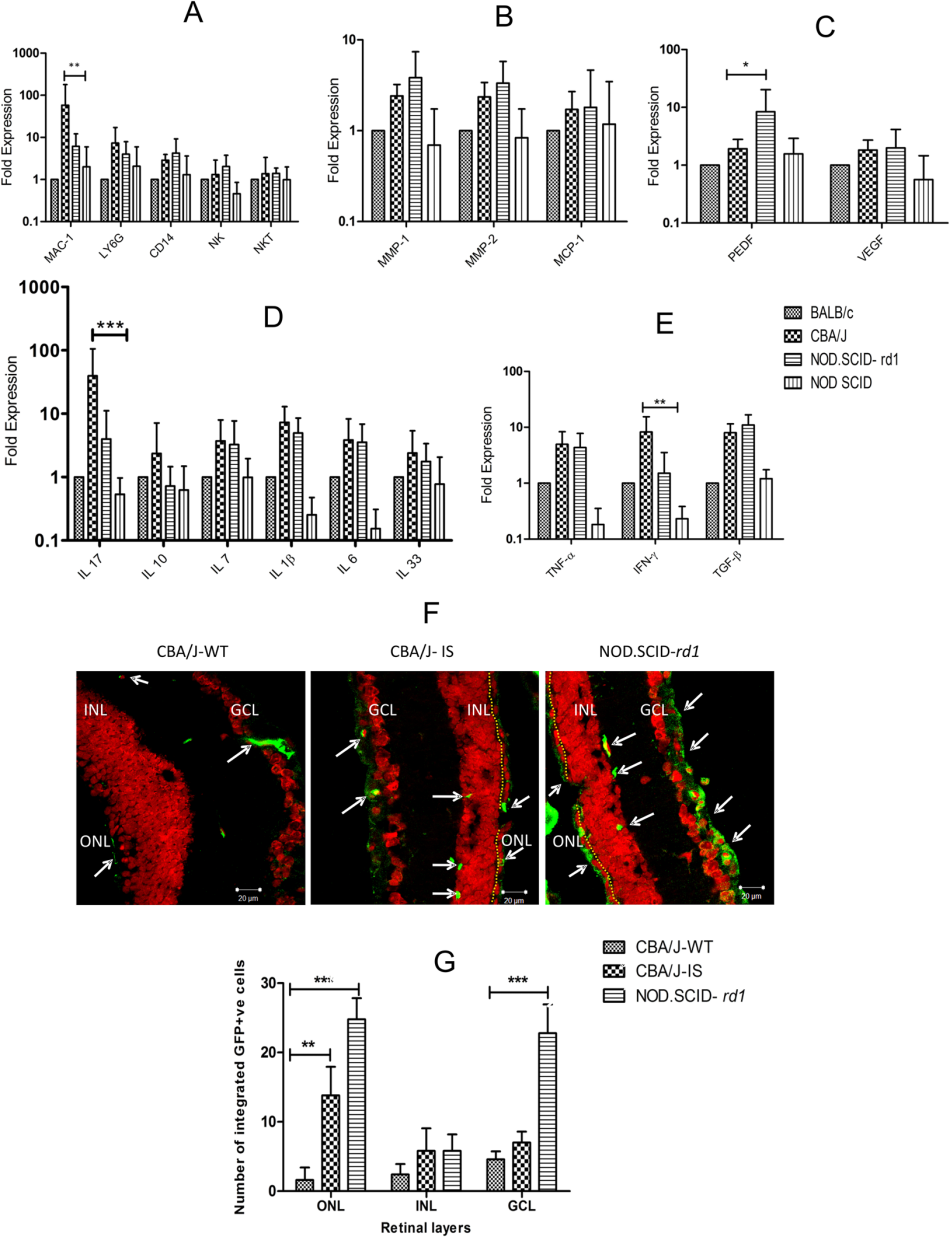
**Effect of immune deficiency on immune privilege and transplantation.** (A) Macrophage marker MAC1 displayed significant upregulation in CBA/J as compared to NOD.SCID-*rd1* mice. Neutrophil marker (Ly6G) was slightly higher in CBA/J and natural killer (NK and NKT) cells were comparable in both CBA/J and NOD.SCID-*rd1*. (B) Pigment epithelial growth factor (PEDF) secreted by RPE cells was considerably elevated in NOD.SCID-*rd1* as against CBA/J. There was no significant change in the expression of vascular endothelial growth factor (VEGF) between CBA/J and NOD.SCID-*rd1*. (C) Matrix metalloproteases (MMP1 and MMP2) and monocyte chemoattractant protein (MCP1) were upregulated in both CBA/J and NOD.SCID-*rd1* as compared to control, however there was no significant difference between the two groups. (D) The proinflammatory interleukins (IL-17, IL-7, IL-1b, IL-6 and IL-33) were increased in both CBA/J and NOD.SCID-*rd1* mice while there was no observed change in IL-10 expression in NOD.SCID-*rd1* compared to control. CBA/J exhibited significantly high expression of IL-17 compared with NOD.SCID-*rd1*. (E) Another set of pro-inflammatory cytokines (TNF-α, IFN-γ and TGF-β) also indicated upregulation in both RD models; however, IFN-γ expression was considerably higher in CBA/J. (F) GFP^+^ mouse retinal cells (1×10^6^) were transplanted subretinally in the eye of three groups of mice (4 weeks): CBA/J- wild type (CBA/J-WT), CBA/J-Immune suppressed (CBA/J-IS) and NOD.SCID-*rd1*. The representative confocal images (63×) of retinal sections are shown here. GFP^+^ cells integrated into the retina of all the three groups (arrows indicate the retinal layers where cell engraftment has occurred); however, CBA/J-WT exhibited least engraftment with a few cells integrating into GCL and still fewer cells in INL. CBA/J-IS mice showed significant cell integration into GCL and a few cells in INL. A single line of ONL, seen above yellow dotted line, was also preserved and stained GFP^+^, suggesting that donor photoreceptor cells survived and integrated into host CBA/J-IS retina. NOD.SCID-*rd1* exhibited maximum cell integration amongst the three groups of animals. GFP^+^ cells integrated in high numbers in GCL and a few in INL. However, like CBA/J-IS, it also showed donor GFP^+^ photoreceptor cell integration just above INL in significant numbers. (G) Graphical representation of cell engraftment quantification in the three major layers of retina (ONL, INL and GCL) in each group of animals and their comparison to characterize potential cell-based therapeutics of each group. ***P<0.001, **P<0.01 and *P<0.05.

Factors involved in maintenance of immune privilege in the eye were checked by quantitative PCR analysis in wild-type CBA/J, immunosuppressed CBA/J and NOD.SCID-*rd1* group of animals. Wild-type CBA/J and NOD.SCID-*rd1* models exhibited upregulated proinflammatory markers like IL-7, IL-10, IL-1B, IL-33, TNF-α, TGF-β and IFN-γ. The expression of macrophage markers (CD14 and MAC-1), neutrophils (Ly6G), monocyte chemoattractant protein (MCP-1), angiogenesis marker (VEGF) and matrix metalloproteases (MMP-1 and MMP-2) were also upregulated in retinal degeneration models CBA/J and NOD.SCID-*rd1*. However, CBA/J suffered significantly higher macrophage infiltration as well as considerably upregulated IFN-γ and IL-17 secretion as compared to NOD.SCID-*rd1*. Surprisingly, the level of pigment epithelium-derived factor (PEDF) involved in photoreceptor cell survival and retinal viability was highly upregulated in NOD.SCID-rd1 but not in CBA/J (Fig. 7A-E).

### Cell transplantation studies to affirm the use of NOD.SCID-*rd1* for potential therapeutic purposes

The three groups of animals were analyzed for GFP-positive retinal cell engraftment post-transplantation by immunofluorescence studies. We found that all the three groups of animals showed cell engraftment, of which the NOD.SCID-*rd1* mouse model exhibited maximum engraftment. The engraftment of cells in the GCL was observed in all the groups but a substantial number of cell integration in the INL and ONL was visible only in NOD.SCID-*rd1* (Fig. 7F,G).

## DISCUSSION

In this study, a new immunocompromised RP mouse model has been developed, characterized and analyzed for potential therapeutic cell-based transplantation studies for the restoration of vision during RP caused due to *Pde6b* mutation. Around 2-5% of autosomal recessive RP (arRP) in humans is due to *PDE6B* mutation, which leads to severe photoreceptor degeneration (Ferrari et al., 2011). Extensive cell-based therapies to restore visual function during RP are already in progress that employ xenogenic cell sources to derive photoreceptor progenitors or photoreceptor cells which use either immunocompetent RP mice models considering the immune privilege of the eye (Assawachananont et al., 2014; Kamao et al., 2014; Li et al., 2016) or immunosuppressant to eliminate chances of immune rejection (Tezel et al., 2007). However, none of these methods seem efficient enough to produce an unbiased observation. Cells transplanted in immunocompetent models can still face immune rejection or immune modulation (Lund et al., 2003) while long-term immunosuppressant usage may cause negative impact on the health of recipient (Thliveris et al., 1991). Immunodeficient mouse models are also frequently used to demonstrate xenogenic cell transplantation (Decembrini et al., 2014). Even so, the cell transplantation studies in immunodeficient mice with normal vision do not mimic the degenerated state of retina.

Since RP is considered a hereditary disease, the role of immune cells in the progression of retinal degeneration remains unexplored. Inflammatory processes due to a breach by the immune system have long been implicated in the pathogenesis and sequelae of various ocular diseases like noninfectious uveitis and macular edema (Camelo, 2014; Mochizuki et al., 2013; Zhou and Caspi, 2010). Recent results also support a prominent role for inflammation underlying the pathogenesis of a wide array of retinal diseases, including age-related macular degeneration (AMD) (Kauppinen et al., 2016), diabetic retinopathy (DR) (Kastelan et al., 2007; Semeraro et al., 2015), retinal vein occlusion (RVO) (Karia, 2010) and retinitis pigmentosa (RP) (Whitcup et al., 2013). Lymphocytes have been found in the vitreous gel of RP patients, thus further characterizing an inflammatory nature of vitreous cells, hinting at a crucial role played by immune cells in the RP set-up (Whitcup et al., 2013).

Seiler et al. recently developed a nude pigmented retinal degenerated rat strain that lacks T cells to study transplantation of human cells (Seiler et al., 2014). Our NOD.SCID-*rd1* mice provide an improved, well characterized and robust immunocompromised rd1 model that lacks adaptive immunity (T- and B-cells) along with reduced NK cell function. Therefore, these animals can also provide insights into the role of immune cells during pathogenesis of RP.

Elaborate characterization of the NOD.SCID-*rd1* mouse model was performed in order to determine the changes, if any, that are exhibited due to the immunocompromised state of mice during retinal degeneration. Hematological studies revealed that WBC count in NOD.SCID-*rd1* mice was highly reduced compared with CBA/J. The lymphocyte count (m/mm^3^) in NOD.SCID-*rd1* was comparable to NOD SCID mice and lower than CBA/J. However, the monocyte and granulocyte count of NOD.SCID-*rd1* mice was in the same range as CBA/J while NOD SCID showed an increased count for both the parameters. BALB/c was a normal vision control (lacking both the mutations). Other parameters, like hemoglobin, MCH, MCHC and hematocrit were found within range and comparable in all the three strains as compared to BALB/c.

Based on the flow cytometric analysis, we confirmed the presence of a minimal proportion of immune cells (T cells, B cells and NK cells) in the peripheral blood of NOD.SCID-*rd1* mice. However, in spleen, the level of CD3^+^, CD4^+^, CD8^+^, B220^+^ and NKT^+^ immune cells showed no significant change in proportion between CBA/J and NOD.SCID-*rd1* with an exception of CD14^+^, CD11c^+^ and CD8^+^ cells. CD11c^+^ (dendritic cells) and CD14^+^ (monocytes or macrophages) cells, which are an important constituent of innate immunity exhibited significant elevation in NOD.SCID-*rd1*. It is already known that dendritic cells and macrophages may amplify the inflammatory process via cell to cell contact, immune complex formation and complement activation leading to additional RPE cell damage, potentially producing a state of chronic inflammation (Whitcup et al., 2013; Yoshida et al., 2013), indicating their crucial role during progression of retinal degeneration in the absence of adaptive immunity.

On the other hand, NOD.SCID-*rd1* showed a higher proportion of CD3^+^ cells and highly reduced levels of CD8^+^ cells compared with NOD SCID. This result seems quite unusual as NOD SCID mice lack T, B and NK cells in the spleen along with peripheral blood. NOD.SCID-*rd1*, being a cross between CBA/J and NOD SCID, tends to adopt an intermediate proportion of immune cells in the spleen, which may not be essentially functional. To date, the complex interactions between cells and the signals regulating expansion and changes in the composition of various cell types during RP are poorly understood. The NOD.SCID-*rd1* mouse model may thus help to interpret and analyze these differences of immune cells further and understand their absolute role in RP. The relative changes in the immunoglobulin levels of NOD.SCID-*rd1* and NOD SCID mice were not significant as compared to CBA/J, suggesting that B cells in NOD.SCID-*rd1* were lacking or non functional, if present at all.

The histopathological studies of retinal structure revealed that CBA/J and NOD.SCID-*rd1* at 4 weeks of age lost the outer segment of the photoreceptor layer and ONL while BALB/c displayed an intact retina at the same age. The thickness of the INL further reduced at 8-10 weeks of age in CBA/J and NOD.SCID-*rd1* while the retinal thickness of BALB/c increased with time, indicating maturation. This further confirmed that NOD.SCID-*rd1* demonstrated almost similar retinal degeneration kinetics as CBA/J. The q-PCR analysis also suggested loss of rod cells and an increase in the expression of both glial cell markers and amacrine cell markers, indicating that these cells may play a crucial role during retinal degeneration (Fernández-Sánchez et al., 2015).

Similar results were observed upon apoptosis studies by TUNEL assay in CBA/J and NOD.SCID-*rd1*at 4 weeks of age to analyze the effect of photoreceptor cell loss on other retinal cell types. The results revealed that the INL and GCL in CBA/J and NOD.SCID-*rd1* retina exhibited severe apoptosis after the photoreceptor layer was completely degenerated. Since the rod cells form 95% of the photoreceptor layer, their complete loss causes broken contact between RPE layer and cone cells. The RPE layer transfers all the nutrition required by the retina through the blood-retina barrier. This loss of contact may leave the cone cells starving and cause apoptosis in cone photoreceptor cells, bipolar cells, horizontal cells and ganglion cells. The bipolar cell apoptosis may also be caused due to a mutation already present in the rd1 mouse model (*Gpr179^−/−^*), as reported earlier (Nishiguchi et al., 2015).

While both CBA/J and NOD.SCID-*rd1* displayed elevated levels of apoptosis in their respective retina as compared to BALB/c, NOD.SCID-*rd1* exhibited significantly lower levels of apoptosis than CBA/J, suggesting that regardless of photoreceptor cell loss in both the rd1 strains, NOD.SCID-*rd1* shows a much slower progression of apoptosis in other retinal cell types than CBA/J. This observation may be explained by the absence of adaptive immunity in NOD.SCID-*rd1*, which might have a role in apoptosis of retinal cells after breaching into posterior chamber of eye during RP.

In rd1 mice, all rods die by ∼1 month of age. Not surprisingly, the wild-type retina showed robust rhodopsin and opsin expression. Interestingly, we also found rhodopsin expression in the INL and GCL of NOD.SCID-*rd1*mouse retina, but not in CBA/J. It has been reported that Müller glial cells express rhodopsin and opsin as they are considered to have stem cell properties that generate the lost retinal cells (Ooto et al., 2004; Osakada et al., 2010). However, since the CBA/J retina is devoid of any marker expression per se, the exceedingly high rate of apoptosis that leads to death of other retinal cell types may account for the absence of rhodopsin and opsin-expressing Müller glial cells.

Western blot analysis results reconfirmed the loss of rod photoreceptor cells. However, reduced protein expression of bipolar cell marker (PKC-α) was noticed in both CBA/J and NOD.SCID-*rd1*. An increased expression of glial cells expressing GFAP and amacrine and/or ganglion cells expressing CRALBP was also observed. The result concurs with recent reports that suggest there is a marked increase in the expression of Müller glial cells as a neuroprotective mechanism owing to the degeneration and apoptosis of the retinal cells (Francke et al., 2005; Roesch et al., 2012).

In the fundoscopic examination, BALB/c displayed a healthy fundus devoid of any patched structures or lobules whereas CBA/J showed a patched fundus and thick attenuated and sclerotic retinal vessels, which was less severe in NOD.SCID-*rd1* with less obvious patches and visible retinal vessels. This may be due to lack of adaptive immune system which might play a role in the progression of the disease by breaching in after the loss of PR layer. NK cells and NKT cells also co-ordinate with several key players of innate immunity involved in retinal degeneration and its after-effect cascades (Bharti et al., 2014b). Thus, the absence of both adaptive immunity and NK/NKT cells in NOD.SCID-*rd1* might have caused slower degeneration and attenuation.

ERG characterization of the NOD.SCID-*rd1* mice was performed against CBA/J and BALB/c mice as positive and negative controls for RP, respectively. The equally altered a-wave, which suggests an absence of signals from photoreceptor cells on stimulation with flashed light in CBA/J and NOD.SCID-*rd1* concurred with other observations indicating loss of photoreceptor cells at around 4 weeks of age. While CBA/J also displayed a highly diminished b-wave as compared to BALB/c, NOD.SCID-*rd1* exhibited a significant preservation in the b-wave, suggesting retinal cell types other than photoreceptor cells were active, functional and more viable as compared with CBA/J.

Behavioral observations at the age of 4-6 weeks to validate the vision loss were further analyzed to comprehend the molecular and functional changes that occurred during RP. The visual cliff test indicated a loss of depth perception ability of both CBA/J and NOD.SCID-*rd1* strains suffering from retinal degeneration during RP owing to their inability to differentiate between the shallow and deep sides of the cliff. Not surprisingly, CBA/J and NOD.SCID-*rd1* lack optokinetic response in view of retinal degeneration that depletes them of ability to differentiate between two objects during movement. Interestingly BALB/c also displayed a limited response towards 0.03 and 0.13 cpd while a lesser response was seen for 0.26, 0.52 and none towards 1.25 cpd gratings. It has been reported that albino animals display alterations of the visual system due to the absence of melanin from the retinal pigment epithelium, which is responsible for scattering the light between directions of sight (Abdeljalil et al., 2005). The light/dark latency test performed to analyze the aversion of mice to light (as they are nocturnal animals), risk-taking behavior and exploratory behavior conveyed that loss of vision in CBA/J and NOD.SCID-*rd1* causes lowered aversive behavior towards light and also lowers the risk-taking behavior of these mice as compared to BALB/c. The number of transitions was significantly higher in BALB/c than CBA/J or NOD.SCID-*rd1* suggesting that the exploratory behavior of mice with retinal degeneration is highly down regulated.

The qPCR analysis of immune privilege markers in eye of rd1 models indicated that most of the pro inflammatory markers displayed a common upregulation, CBA/J expressing slightly higher levels than NOD.SCID-*rd1*. However, expression of MAC-1 (macrophages) and IFN-γ (inflammatory marker) was significantly higher in CBA/J, which has been reported to cause retinal cell death (Husain et al., 2014). Considerable alleviation was found in expression of PEDF in CBA/J, a growth factor involved in neuroprotection and antiangiogenic actions that supports photoreceptor survival (Tombran-Tink and Barnstable, 2003). Furthermore, cell transplantation studies revealed that cell engraftment was significantly higher in NOD.SCID-*rd1* as compared to WT and IS-CBA/J. Moreover, cell transplantation was distributed in both INL and GCL in NOD.SCID-*rd1* compared with WT and IS-CBA/J. GFP-expressing cells were also observed to form a sheet of ONL in NOD.SCID-*rd1* while a few cells were also seen in IS-CBA/J. This suggests that immune privilege in NOD.SCID-*rd1* although compromised, is not as poor as in CBA/J and therefore the transplanted cells may survive better in this model. Also, immune suppression is not efficient enough to avoid the breaching of retina by immune cells during retinal degeneration when pro-inflammatory signals flare up and recruit systemic immune cells. It is also likely that immune deficiency in NOD.SCID-*rd1* mice helps in sustenance of immune privilege better than immunocompetent CBA/J during retinal degeneration, making NOD.SCID-*rd1* a comparatively better model for cell-based therapeutics.

In conclusion, the initial characterization of NOD.SCID-*rd1* mice model of RP shows promising future directions to explore new arenas in the development and progression of RP. It can be used as a humanized rd1 mouse model, opening new approaches for cell-based treatment trials and study of immunological aspects during retinal degeneration. In addition, the cell therapy techniques in this immunocompromised rd1 mouse model will give real insight into any variability observed in the integration and functionality of cells with or without adaptive immunity which can also help us determine the absolute cell density required for revival of the PR layer. The potential limitations of this model are that it mimics only a single aspect of retinal degeneration (i.e. RP caused by *PDE6B* gene mutation). The immunocompromised state of the model system indicates only the lack of adaptive immunity (T cells and B cells). Therefore, this model can only be used to study for the role of adaptive immunity in immune privilege and consequently in RD during rd1 conditions. In summary, the NOD.SCID-*rd1* mouse model is a useful animal model for therapeutic trials and mechanism dissection of immune regulation during RP.

## MATERIALS AND METHODS

### Animal housing and breeding

NOD.CB17-Prkdcscid/J (NOD SCID), CBA/J, BALB/cByJ (BALB/c) and transgenic C57/B6-GFP [C57BL/6-Tg (UBC-GFP) 30Scha/J] mice were obtained from the Jackson Laboratory, USA. Animals were maintained as per ethical guidelines (CPCSEA). Breeding strategy to obtain double homozygous mutant phenotype is described in detail in supplementary Materials and Methods.

### Hematology

For hematology analyses, 100 μl blood was drawn from the retro-orbital plexus under ketamine-xylazine anesthesia (80 mg/kg and 10 mg/kg body weight). The collected blood was dispensed in the tube containing EDTA, and within 10 min of collection, the samples were analyzed using automated veterinary hematology analyzer MS 4e automated cell counter (MeletSchloesing Laboratories, France) according to the manufacturer's instructions.

### Isolation of crude DNA by high-salt method

DNA was isolated from tail biopsies by salt precipitation using TNES buffer as described earlier (Demayo et al., 2012). The details are given in supplementary Materials and Methods.

### Genotyping for screening of homozygous double mutant phenotype

Genotyping of mice was done for *Pde6b* by SNP-RFLP (single nucleotide polymorphism-restriction fragment length polymorphism) and for *Prkdc* gene by PCR-CTPP (polymerase chain reaction-confronting two primer pairs) (Maruyama et al., 2002). Details are given in supplementary Materials and Methods.

### Immune cell analysis in NOD.SCID-*rd1* mice

Flow cytometry was used to evaluate the proportion of immune cells (CD3, CD4, CD8, B220 and NKT cells) in the peripheral blood and spleen of parent NOD.CB17-Prkdcscid/J mice, parent CBA/J mice, as well as the NOD.SCID-*rd1* mice (*n*=15 for each group). The detailed steps are available in supplementary Materials and Methods.

### Relative quantification of immunoglobulin secretion

Serum was obtained from 4- to 6-week-old NOD SCID, CBA/J and NOD.SCID-*rd1* animals (*n*=10 for each group) to perform the relative quantification of immunoglobulins (IgG1, IgG2a, IgG2b, IgG3, IgM, and IgA). ELISA was performed as per manufacturer's instruction (BD mouse immunoglobulin isotyping ELISA kit). Details are provided in the supplementary Materials and Methods.

### Qualitative analysis of spleen

The animals from different groups were sacrificed and spleen was dissected out and qualitative comparison of the size of spleen was performed.

### Quantitative RT-PCR analysis for retina-specific genes and immune privilege markers

qPCR was performed to check the expression of retinal markers and immune privilege markers in NOD.SCID-*rd1* as compared to CBA/J. Details are given in supplementary Materials and Methods. The expression of genes was normalized by the housekeeping gene (18S rRNA) and relative expression was calculated using the ΔΔCt method.

### Western immunoblotting analysis

Protein lysates were prepared from eye samples by homogenization, quantified by using bicinconinic acid (BCA) kit (Pierce, Rockford, IL) and western blot analysis was performed. The details are given in supplementary Materials and Methods.

### Immunocytochemistry analysis of photoreceptor cells

Immunocytochemistry was performed for detection of Rhodopsin (Thermo Fisher Scientific, MA-1-722, 1:200); S-opsin (Novos Biologicus, NBP-1-20194, 1:200) as rod and cone retinal cell-specific markers, respectively, using standard techniques. Representative confocal photomicrographs were captured at 63× magnification using a system incorporated in the microscope (Zeiss LSM v. 4.2.0.121). Detailed steps are given in supplementary Materials and Methods.

### Histopathological analysis of retinal degeneration during RP

Whole eye was enucleated after sacrificing the animals by cervical dislocation. The tissue was fixed overnight in 10% formalin and embedded in paraffin blocks. Tissue sections of 4 μm thickness were obtained on poly-L-lysine (Sigma-Aldrich)-coated slides using a microtome and stained with Hematoxylin and Eosin using standard techniques. Representative photomicrographs were captured at a 20× magnification using a system incorporated in the microscope.

### Fundoscopic retinal imaging

Fundoscopy was performed for both eyes of anaesthetized animals and images were captured using Streampix software and a MICRON III rodent imaging system (Phoenix Research Labs). The details are provided in supplementary Materials and Methods.

### Electroretinography

Focal ERG was performed according to ISCEV guidelines using Micron III rodent retinal imaging system (Phoenix Research Labs, USA). ERGs were recorded simultaneously from both eyes to examine the retinal function. The ERG responses were obtained through ERG attachment of MICRON III rodent imaging system using Labscribe software (Phoenix). The details are provided in supplementary Materials and Methods.

### Terminal deoxynucleotidyltransferase dUTP nick end labeling (TUNEL) assay for the analysis of apoptosis in RP

Apoptosis in the retina was evaluated through TUNEL assay using the Dead End Fluorimetric TUNEL System (Promega, USA), as per the manufacturer's recommendation. Details are given in supplementary Materials and Methods.

### Behavioral analysis

Behavioral tests were performed to analyze for depth perception and visibility (visual cliff test), aversiveness towards light and exploratory behavior (light/dark latency test) and optokinetic response (optokinetic drum) (Schmucker et al., 2005) in mice of different groups. The details are given in supplementary Materials and Methods.

### Cell transplantation in the retina and post-transplantation engraftment analysis

GFP positive retinal cells (1×10^6^) were transplanted in the eye of each group of animals (WT-CBA/J, IS-CBA/J, NOD.SCID*-rd1*). The animals were euthanized 48 h post transplantation and eyes were isolated, processed and sectioned. The sections were further stained with anti-GFP antibody (Santa Cruz, sc-101525, 1:100) and PI (for nucleus) and representative images were captured at 63× magnification by confocal microscope (Zeiss LSM v.4.2.0.121). Cell engraftment was calculated by counting the GFP-stained cells in five fields for ONL, INL and GCL separately in each animal group using ImageJ software. Details are given in the supplementary Materials and Methods.

### Statistical analysis

The results are presented as mean±s.d. We determined the statistical significance of differences between two groups using one-way ANOVA with Bonferroni *post hoc* test or two-way ANOVA test. The value of *P*<0.05 was considered significant. The statistical analysis was performed using Graph Pad Prism software (v.5.04).
